# Closed Rupture of the Extensor Tendon of the Ring and Little Fingers Following Plate Fixation for Distal Ulna Fracture: A Case Report

**DOI:** 10.7759/cureus.103679

**Published:** 2026-02-15

**Authors:** Tatsurou Imai, Mineyuki Zukawa, Tatsurou Hirokawa, Yoshiharu Kawaguchi

**Affiliations:** 1 Orthopaedics, University of Toyama, Toyama, JPN

**Keywords:** distal ulna fracture, metallosis, plate fixation, tendon rupture, walant

## Abstract

Extensor tendon rupture following plate fixation for distal ulna fractures is extremely rare. We present a case of delayed closed rupture of the extensor tendons of the ring and little fingers following distal ulna plate fixation. A 71-year-old woman developed progressive swelling and loss of active extension of the ring and little fingers eight years after surgery for distal radius and ulna fractures. Computed tomography demonstrated dorsal penetration of the most distal ulnar screw. Surgical exploration confirmed rupture of the extensor digitorum communis tendons to the ring and little fingers and the extensor digiti minimi tendon. Screw removal and end-to-side tendon transfer were performed, resulting in satisfactory functional recovery without rerupture. This case suggests that dorsal screw prominence in distal ulna plating may contribute to delayed attritional extensor tendon rupture. Surgeons should carefully assess screw length intraoperatively and consider early implant removal when tendon irritation is suspected.

## Introduction

Rupture of the flexor pollicis longus (FPL) tendon following volar plate fixation for distal radius fractures is a well-documented phenomenon [1]. Various mechanisms have been proposed to explain, including tendon ischemia and malunion-related deformity. However, mechanical impingement between the tendon and the volar plate is considered to be the most common cause [2]. Tendon reconstruction using the palmaris longus tendon is commonly performed in such cases [2]. It is important to note that FPL tendon rupture is often recognized as a delayed complication that may occur several years after the initial surgery.

In recent years, plate fixation has been performed for distal ulna fractures associated with distal radius fractures, with favourable outcomes reported [3].

In contrast, to our knowledge, there have been no reports of tendon rupture following plate fixation for distal ulna fractures. Here, we present a rare case of subcutaneous rupture of the ring and little finger extensor tendons after plate fixation for a distal ulna fracture. This was successfully treated with tendon transfer.

## Case presentation

A 71-year-old woman developed swelling in her dorsal wrist without any apparent trigger and was unable to extend her right ring and little fingers. Eight years earlier, she had undergone plate fixation for distal radius and ulna fractures at another institution.

Physical examination

**Figure 1:**
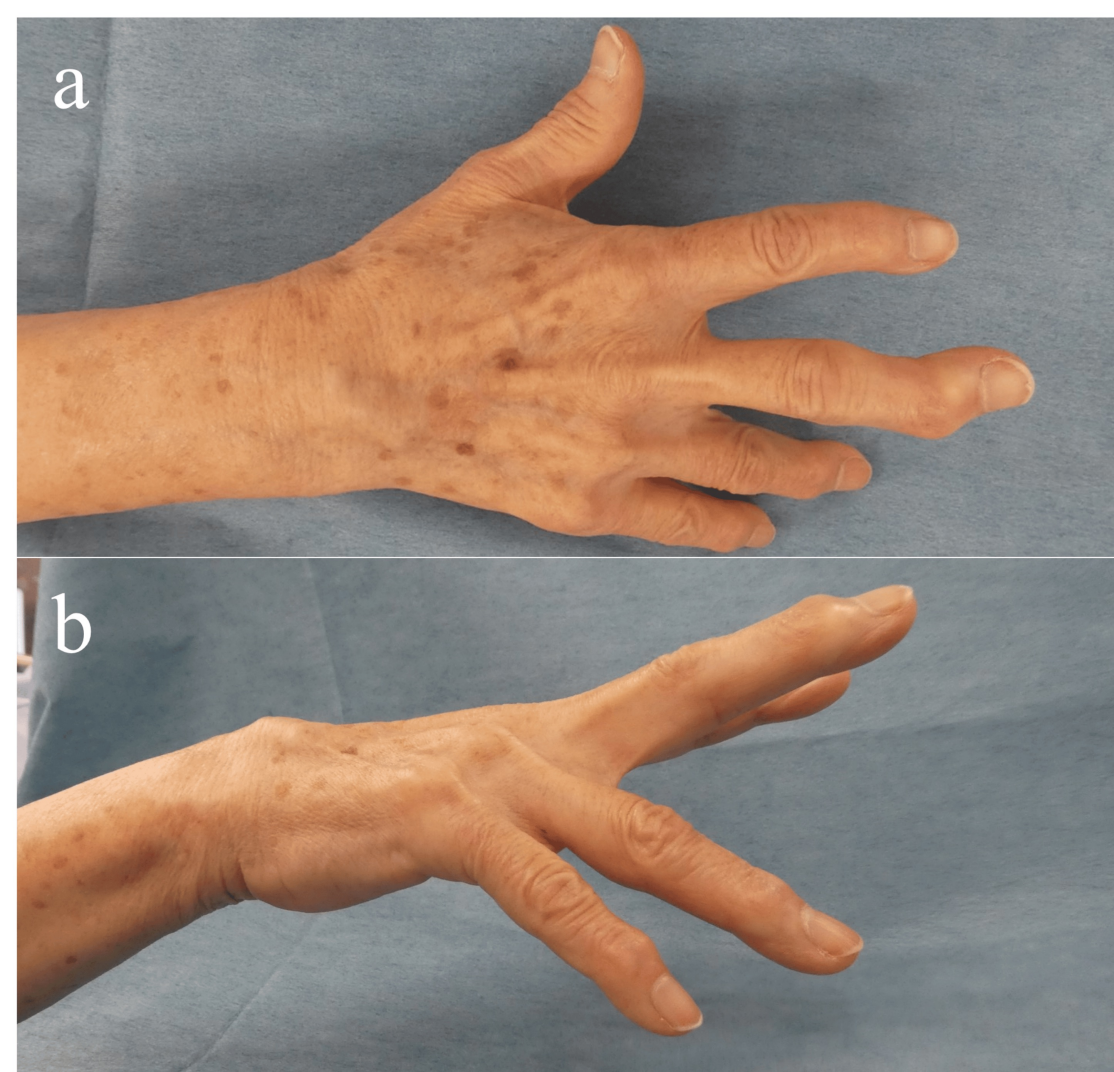
Clinical photographs of the right hand: (a) dorsal view of the hand; (b) ulnar aspect of the hand. (a-b) Extension lag of the ring and little fingers and swelling in the fourth and fifth dorsal compartments.

Forearm range of motion was 60° of supination and 70° of pronation. Wrist range of motion was 40° of dorsiflexion and 40° of palmar flexion. Metacarpophalangeal joint active extension was 0° in the index and middle fingers, -36° in the ring finger, and -46° in the little finger, indicating extension lag of the ring and little fingers, which interfered with activities of daily living such as washing her face. Swelling was noted distal to the fourth and fifth dorsal compartments (Figure 1).

Imaging

**Figure 2:**
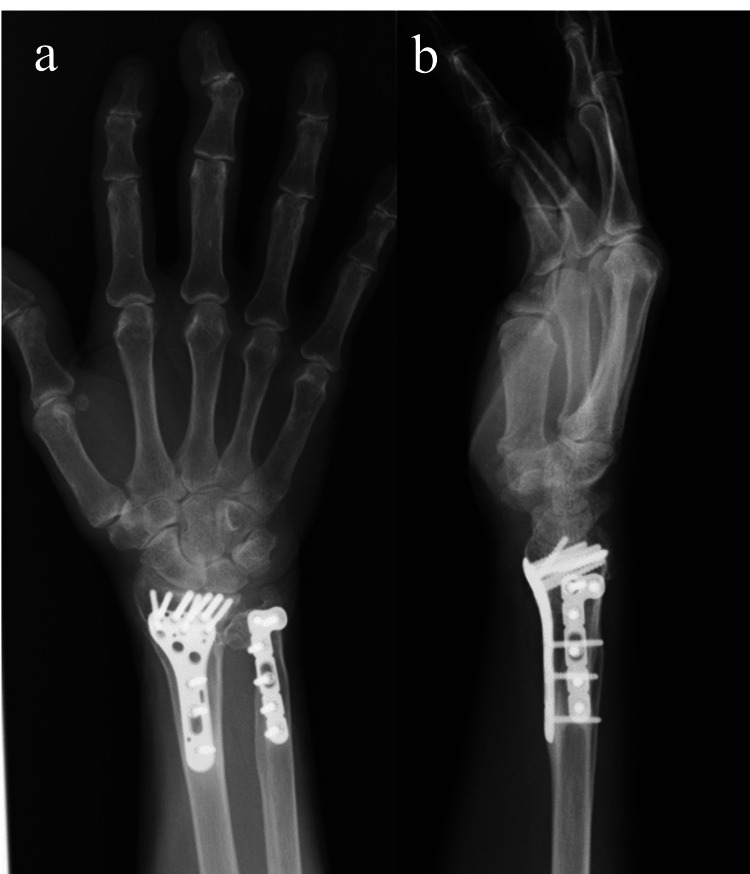
Posteroanterior (a) and lateral (b) radiographs after plate fixation for distal radius and ulna fractures. Bony union has been achieved; however, osteoarthritic changes of the distal radioulnar joint (DRUJ) are present.

Plain radiographs demonstrated bony union of both the radius and ulna fractures, with degenerative changes in the distal radioulnar joint (Figure 2).

**Figure 3:**
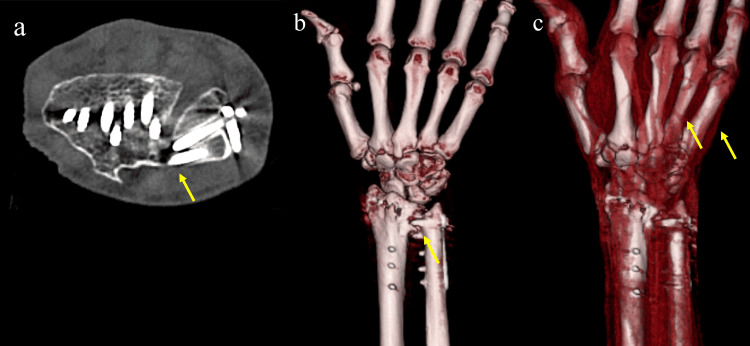
Computed tomography (CT) images: (a) axial CT image; (b) three-dimensional CT image; and (c) three-dimensional CT images of the extensor tendons.

Computed tomography revealed dorsal screw penetration at the distal ulna (Figures 3a-3b). Three-dimensional CT showed bowing and discontinuity of the extensor digitorum communis tendons to the ring and little fingers at the mid-metacarpal level, suggesting extensor tendon rupture (Figure 3c).

Surgery

**Figure 4:**
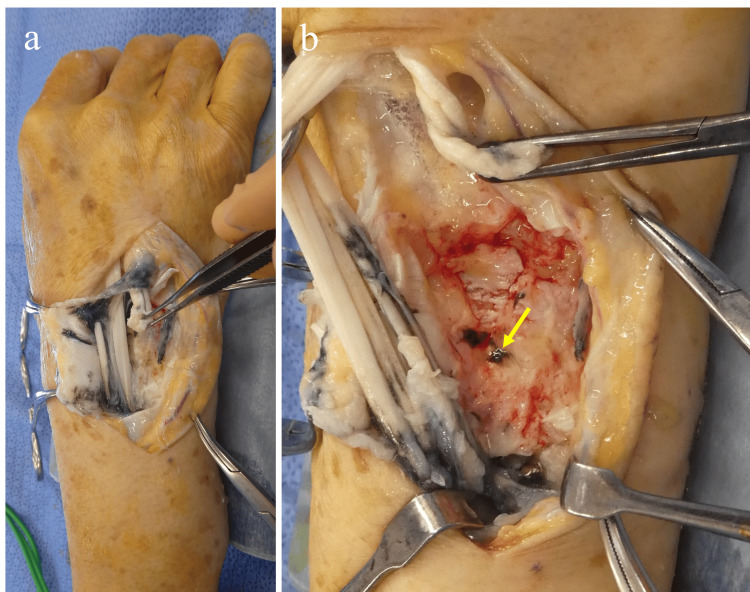
Intraoperative findings. After incision of the extensor retinaculum. (a) Proliferation of blackened synovium suggestive of metallosis is observed. (b) The distal ulnar screw is seen penetrating dorsally with associated rupture of the dorsal joint capsule.

Using wide-awake local anaesthesia with no tourniquet (WALANT) technique, screw removal and end-to-side tendon transfer were performed. After releasing the extensor retinaculum, dorsal capsular rupture caused by the most distal screw and blackened synovial membrane proliferation suggestive of metallosis were observed (Figures 4a-4b).

Following synovectomy, rupture of the extensor digitorum communis tendons to the ring and little fingers (EDC4,5) and the extensor digiti minimi (EDM) tendon was confirmed (Video 1).

**Video 1:** Intraoperative findings demonstrating rupture of the extensor digitorum communis tendons to the ring and little fingers (EDC4 and EDC5) and the extensor digiti minimi tendon.

The screws were removed; however, plate removal was technically difficult, and as there were no symptoms attributable to plate irritation, the plate was left in situ. For reconstruction, end-to-side tendon transfer of EDC4, EDC5, and EDM to the extensor digitorum communis tendon of the middle finger (EDC3) was performed, and improvement in active extension was confirmed intraoperatively (Video 2).

**Video 2:** Tendon transfer of the extensor digitorum communis tendons to the ring and little fingers to the extensor digitorum communis tendon of the middle finger using a side-to-end technique. Improvement of active finger extension was confirmed intraoperatively.

Postoperative course

Early mobilization was initiated with buddy taping just after surgery. At five months postoperatively, full active finger extension was maintained without rerupture.

## Discussion

Distal ulna fractures, excluding ulnar styloid fractures, rarely occur in isolation and are reported to accompany approximately 5% of distal radius fractures [4]. Although fixation of distal ulna fractures is often omitted when stable fixation of the distal radius is achieved, fixation is recommended in cases with persistent instability at the ulnar fracture site or when adequate reduction cannot be achieved [5].

Reported complications following plate fixation for distal ulna fractures include implant irritation requiring removal and ulnar nerve neuropathy [3,6,7]. However, no previous reports have been published regarding tendon rupture associated with distal ulna plates. Extensor tendon rupture after plate fixation for distal radius fractures has been well reported, particularly involving the extensor pollicis longus (EPL) tendon [8]. Proposed mechanisms include dorsal screw penetration, attritional wear over prominent hardware, ischemic changes associated with increased compartment pressure, and synovitis caused by metal debris. In contrast, extensor tendon rupture associated with distal ulna plates has not been previously reported, and the anatomical relationship between the distal ulna and the dorsal extensor compartments differs from that of the distal radius.

In the present case, penetration of the most distal screw through the dorsal joint capsule, resulting in protrusion into the ulnar side of the fourth and fifth compartments, was considered the primary cause of tendon rupture.

## Conclusions

Plate fixation is useful for distal ulna fractures in cases with fracture instability or when adequate reduction is difficult to achieve. However, surgeons should be aware of the potential risk of extensor tendon rupture. If postoperative imaging suggests a high risk of tendon injury, early implant removal should be considered. In addition, occult complications should be detected at an early stage through clinical assessments, including tests such as the EDM test, and treated promptly.
